# Quantitative CCTA Plaque Differences Between Japanese and Non-Japanese Patients: Insights From ADVANCE

**DOI:** 10.1016/j.jacadv.2026.103234

**Published:** 2026-09-15

**Authors:** Alexander Haenel, Fionn Coughlan, John K. Khoo, Nicholas Ng, Hitoshi Matsuo, Hiromasa Otake, Campbell Rogers, Bjarne Linde Nørgaard, Jonathon Leipsic, Georgios Tzimas

**Affiliations:** a Department of Radiology, Royal Columbian Hospital & University of British Columbia, Vancouver, British Columbia, Canada; b Department of Radiology and Nuclear Medicine, University Hospital of Schleswig-Holstein, Lübeck, Germany; c Fiona Stanley Hospital, Murdoch, Western Australia, Australia; d St George Hospital, Kogarah, New South Wales, Australia; e HeartFlow Inc, Mountain View, California, USA; f Department of Cardiology, Gifu Heart Center, Gifu, Japan; g Division of Cardiovascular Medicine, Department of Internal Medicine, Department of Cardiology, Kobe University Graduate School of Medicine, Kobe, Japan; h Aarhus University Hospital, Aarhus, Denmark; i Department of Radiology, St Paul's Hospital & University of British Columbia, Vancouver, British Columbia, Canada; j Lausanne University Hospital and University of Lausanne, Lausanne, Switzerland

**Keywords:** artificial intelligence, coronary atherosclerosis, CT quantitative plaque assessment, Japanese ethnicity, plaque volumes

**Figure:**
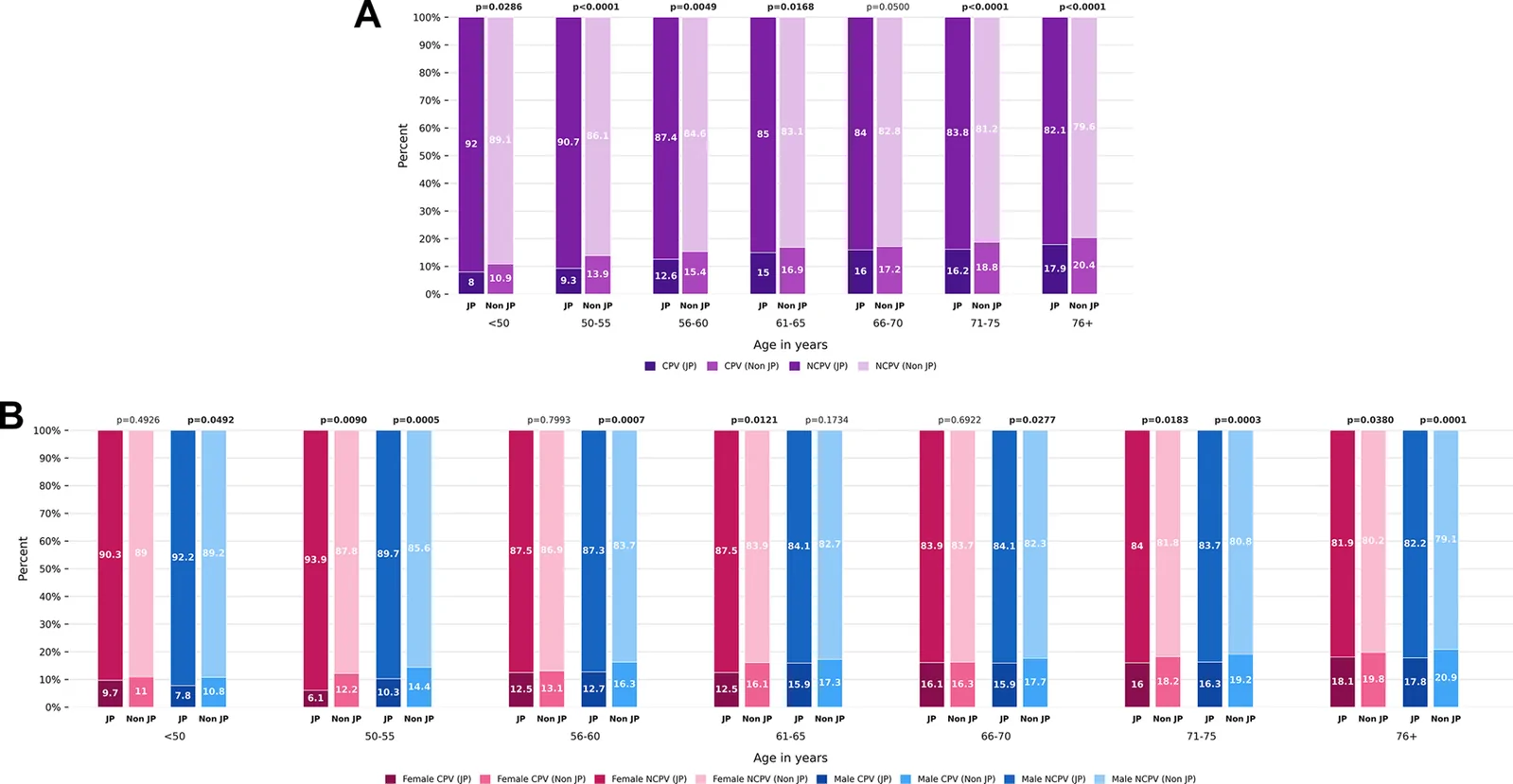

Recently, population distribution percentiles of total plaque volume (TPV) derived from artificial intelligence (AI)-supported analyses were proposed as the most significant metric for cardiac computed tomography (CCTA)-based risk stratification and treatment recommendations.^1^^,^^2^ As ethnicity is a well-established determinant of coronary artery disease prevalence and progression, interest in its impact on TPV has grown.^3^ However, the distribution of TPV and other quantitative plaque metrics in the Japanese population has not been specifically evaluated. This study aimed to compare quantitative plaque metrics derived from CCTA between Japanese and non-Japanese participants from a large, consecutive, multicenter cohort using AI-Quantitative Coronary Plaque Analysis (AI-QCPA).

This retrospective, cross-sectional analysis included participants from the prospective, multicenter, international Assessing the Diagnostic Value of Noninvasive CT-FFR in Coronary Care ADVANCE registry.^4^ From 2015 to 2017, 5,083 clinically stable patients were enrolled across 38 centers (13 in Japan), who underwent CCTA for suspected coronary artery disease. Patients with at least 30% stenosis on CCTA were included. Of the initially recruited patients, 190 were not submitted for fractional flow reserve computed tomography and 156 were deemed unanalyzable, leaving 4,737 with adequate image quality for assessment. Of these, an additional 307 cases were deemed unsuitable for AI-QCPA due to reduced image quality. This left 4,430 cases that were included in this analysis. Quantitative assessment of atherosclerotic plaque volumes was performed using AI-QCPA (HeartFlow) which has been validated against intravascular ultrasound.^1^^,^^5^ AI-QCPA was used to quantify and categorize TPV as calcified (CPV), noncalcified (NCPV), or low-attenuation plaque. The volumes (in cubic millimeters) are reported as medians with IQRs in parentheses. These 4 categories were further stratified by vessel volume (defined as plaque volume/vessel volume × 100) to normalize plaque volume for vessel size. To assess the independent associations of cardiovascular risk factors, sex, and age with plaque volume, multiple linear quantile regression was performed, with plaque volume as the dependent variable and age, sex, diabetes, hypertension, hyperlipidemia, smoking status, stenosis, and Japanese ethnicity as independent variables.

Assumptions for linearity were confirmed via visual inspection of residual plots. Comparisons between 2 groups were performed using Wilcoxon rank sum test for continuous variables and chi-square test for categorical variables. Values of *P* < 0.05 were considered to indicate statistical significance. All statistical analyses were performed using SAS software (version 9.4; SAS Institute).

A total of 4,430 participants were included in the analysis; mean age was 66.2 ± 10.1 years and 1,512 (34.1%) were women. The 1,667 (37.6%) participants enrolled in Japan were older (mean age 69.4 ± 9.9 years vs 64.2 ± 9.7 years; *P* < 0.0001) and had a higher prevalence of hypertension (67.4% vs 55.8%; *P* < 0.0001) and diabetes (32.5% vs 16.4%; *P* < 0.0001) compared to participants enrolled in Europe and North America. Furthermore, anatomical stenosis >50% (78% vs 67.5%; *P* < 0.0001) and fractional flow reserve computed tomography ≤0.8 (70.4% vs 63.2%; *P* < 0.0001) were significantly more common among Japanese participants. No significant differences between groups were observed in smoking status (*P* = 0.5918) or hyperlipidemia (*P* = 0.0556).

After multivariable adjustment for age, sex, cardiovascular risk factors, and anatomical stenosis, median TPV was similar between Japanese (396 mm^3^; IQR: 173-788 mm^3^) and non-Japanese participants (386 mm^3^ [155-750] mm^3^; *P* = 0.1040). In contrast, Japanese participants had higher NCPV (331 mm^3^ [153-641] vs 319 mm^3^ [132-596]; *P* = 0.03) and higher corresponding noncalcified percent atheroma volumes 13% (6.2% to 22.3%) vs 12.2% (5.6% to 20.9%) (*P* = 0.0078). The observed differences for NCPV were more pronounced in female participants (*P* = 0.0377) compared to males (*P* = 0.2017). No difference between groups was observed for calcified plaque volume and low-attenuation plaque volume. The proportions of CPV and NCPV as a percentage of TPV for Japanese and non-Japanese participants by age groups and sex are shown in Figure 1.

**Figure 1:**
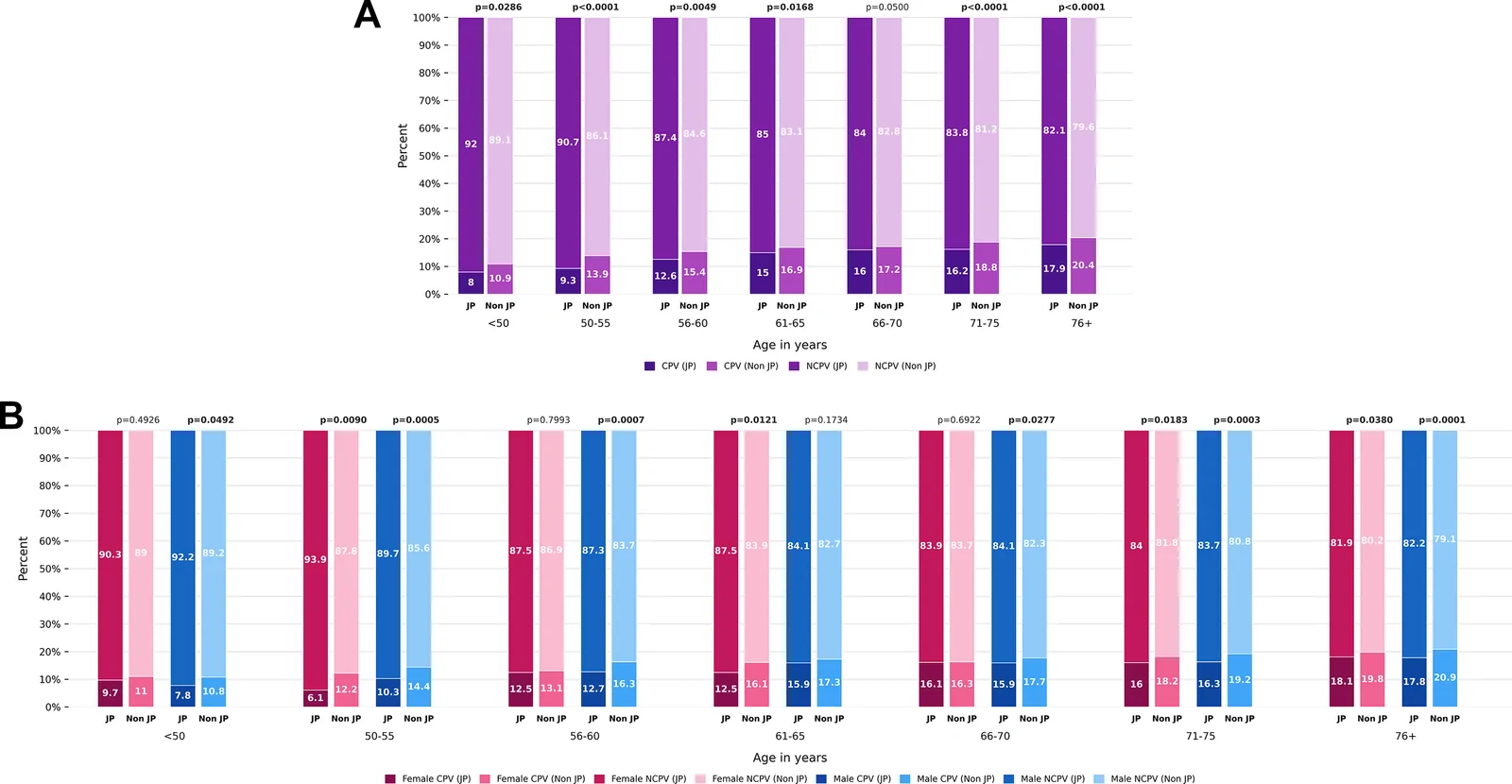
Percentage Coronary Plaque Volumes by Composition and Age in Japanese and Non-Japanese Participants (A) Combined population. (B) Female/male.

In our retrospective analysis of the ADVANCE registry, adjusted TPV was similar between Japanese and non-Japanese participants. This finding may appear surprising, given that East Asians have a lower prevalence of myocardial infarction and cardiovascular mortality than Caucasians.^3^ Although overall plaque burden was similar, Japanese participants demonstrated higher NCPV. Due to the limitations of our analysis mentioned subsequently, further research is needed to clarify whether these findings reflect external factors or true ethnic differences in plaque composition. Our observations may reflect variations in plaque maturation, calcification propensity, and atherosclerosis progression across ethnic groups. This trend was more pronounced in females. This sex-specific pattern potentially reflects the interplay between estrogen-related plaque biology and ethnicity-specific metabolic risk profiles, which together may disproportionately influence plaque composition in women.

Our cross-sectional analysis has limitations, including inherited referral bias in the ADVANCE registry, with only inclusion of participants with documented stenosis of at least 30% which significantly limits extrapolation of results to the general population. In addition, information regarding statin therapy was not recorded. A potentially lower prevalence or intensity of statin therapy in Japanese participants could therefore plausibly have contributed to the modestly higher NCPV observed in this group.

Finally, the aforementioned differences in baseline demographics between groups and heterogeneity in the non-Japanese cohort that includes patients enrolled across Europe and North America, with differing ethnic background, risk profiles and treatment patterns, are additional important limitations.

Further research using AI-QCPA could help to better understand the relationship between plaque composition, ethnicity, and sex.

## Funding support and author disclosures

Dr Leipsic has received consulting fees from Heartflow and Circle CVI; honoraria from Arineta; travel support from Heartflow and Arineta; stock or stock options from Heartflow; and served as deputy editor for Radiology Cardiothoracic Imaging. Mr Ng and Dr Rogers are employees of Heartflow. All other authors have reported that they have no relationships relevant to the contents of this paper to disclose.
